# Research Progress of Cut-Resistant Textile Materials

**DOI:** 10.3389/fchem.2021.745467

**Published:** 2021-09-29

**Authors:** Yanru Zhai, Lizhou Mao, Yue Shen, Xuefeng Yan

**Affiliations:** School of Textile and Garment, Nantong University, Nantong, China

**Keywords:** cut-resistant textile materials, high performance fiber, coating materials, evaluation standard, hand protection

## Abstract

This article describes the physical properties, application fields and modification technologies of several commonly used cut-resistant textile raw materials and coating materials, and summarizes and compares and analyzes the current commonly used cut-resistant textile materials evaluation standards: EN420, EN388, ASTM F-1790, ISO13997. Finally, it is pointed out that lightness, softness and comfort are the future research and development directions of cut-resistant textiles. The article provides a preliminary reference for the application and modification of high-performance fibers and coating materials in cut-resistant textiles.

## Introduction

Nowadays, with the rapid social and economic development, people’s awareness of self-safety protection is constantly improving, the demand for protective equipment is expanding day by day, and the development of protective equipment is accelerated at the same time. As a part of it, the application of protective clothing has gradually entered people’s daily life from the field of professional protection. In short-track speed skating, figure skating and other sports competitions, athletes in the state of high-speed skating are prone to collision. Thus, skates cause cutting injuries to themselves and other athletes from time to time (Wang and Li, 2004). In China, hand injuries account for a large proportion of work-related accidents, accounting for about 25% (Yu, 2018). Compared with other parts of the human body, the hands are more compact and flexible, the nerve endings in the fingers are also more concentrated, and recover more slowly after injury, affecting the normal life of workers. In the production process, there are not a small number of operators who cause cutting injuries due to blades, metal plates and so on. The necessary cut-resistant equipment can help operators effectively avoid operational risks, and can effectively improve production efficiency while reducing the operational threat coefficient. And with the vigorous development of industrial production, people pay more and more attention to the working environment and raise awareness of protecting their own safety and health. Cut-resistant equipment is the last risk control measure to protect the human body. The stronger the anti-cutting ability of the protective equipment, the lower the risk of people suffering from cutting injuries, which can effectively improve productivity while also improving the safety factor.

In order to achieve the protective performance of cut-resistant clothing, it is necessary to clarify that the anti-cutting performance of fabric is mainly achieved through the cut-resistant performance of yarn and fabric. The fabrics commonly used in the field of cut-resistant textile materials mainly include knitted fabrics, non-woven fabrics, three-dimensional fabrics and composite fabrics. The main damage mechanism of the fabric is the shearing of the fabric along the cross section by the cutter and the tensile fracture of the yarn along the axial direction. For single-layer weft knitted fabrics, when the knife tip pierces the knitted fabric loop, due to the loop length transfer characteristics, the length of the loop pierced by the knife tip transfers to the adjacent loop length under the action of a small force. As the penetration width of the tool increases, the length of the coil transfers to a certain extent and no longer transfers. The critical state at this time is called the self-locking state. At this point, the knife edge begins to cut the coil. This self-locking feature can wrap the knife tip to a certain extent to prevent cutting; for double-layer and multi-layer weft knitted fabrics, when the knife tip touches the fabric, the fabric will bend first, and the tool will affect the fabric at this stage. The impact force of the knife is very small. As the knife falls, the knife tip gradually compresses the fabric, and the resistance on the knife tip increases. As the tip of the knife continues to fall, the tip of the knife pierced into the loop becomes wider, the loop is transferred to a certain extent and then it locks itself, the fabric begins to physically deform, the yarn itself stretches and the yarn in contact with the blade is cut and impacted. When the force reaches the maximum value, part of the fabric is pierced, and the uncut yarns of the lower fabric are stretched and deformed; the cutting resistance of non-woven fabrics is far less than that of knitted axial fabrics. In non-woven fabrics, the arrangement of fibers is disordered. When the blade cuts the fabric, the fiber has high breakability at different times; the cut resistance of the composite fabric is an optimization of the cut resistance of various single-structure fabrics. The straightening state of the yarn in the fabric is conducive to the transmission of shock waves in the plane, and the energy absorption area of the fabric is enlarged. The bundling phenomenon of the lining yarn makes it have strong cutting resistance.

Improving the bending and tensile properties of the fabric can effectively absorb the failure energy contained in the external impact, thereby improving the cut-resistant and other protective properties of the fabric (Guo, 2005). From the cutting and destruction process of the woven fabric. It can be seen that compact fabrics have better cut-resistance than loose fabrics. When preparing cut-resistant clothing, the tight fabric structure such as plain weave and twill should be preferred. Because different fiber materials have different properties, the ability of fiber materials itself to resist external impact also determines the anti-cutting performance of textile materials to a certain extent. In the composite fabrics, the polymer/filler ratio also affects the rheological properties of the composite fabrics, and further affects the mechanical properties of the composite materials such as stiffness, modulus, and elongation at break (Ganguly et al., 2019). In addition to improving the bending and tensile properties of fabrics, the modification of natural fibers and the development of high-performance fibers are also the key to enhance the anti-cutting performance of textile materials. Therefore, the research on cut-resistant textile materials is of great significance.

## The Development of Cut-Resistant Textile Materials

Whether natural fiber or synthetic fiber is an important component of cut-resistant textiles, and has always occupied a very important position in the development of national economy and the construction of national defense. The properties of the selected fiber materials have a great influence on the anti-cutting performance of textiles. Generally speaking, the greater the risk faced by the operator, the higher the requirement for the protective performance of the fiber (Yan and Hao, 2012). According to different operation scenarios, it is particularly important to reasonably select fiber materials with different anti-cutting performance levels. The cut-resistant textiles made of traditional natural fiber materials have low protective performance, showing weak mechanical properties and weather resistance, which limit their application in different fields of protective equipment, but their utilization rate is high, light weight, good flexibility and low price, it is still a commonly used protective material in factory production operations (Ghosh et al., 2018; Ghosh et al., 2019). Due to its excellent mechanical strength, good thermal stability and abrasion resistance, high-performance fiber has gradually replaced traditional fiber materials for cut-resistant textile materials. At present, several kinds of high-performance fibers commonly used in cut-resistant textile materials mainly include: aramid 1414, ultra-high molecular weight polyethylene fiber, P-benzoxazole polyester fiber, glass fiber, metal fiber, etc.

### Traditional Anti-Cutting Fiber Materials

At first, people were not very aware of safety protection. Workers in traditional factories were equipped with cut-resistant clothing and textile materials that were mostly natural fibers and synthetic fibers, such as cotton, hemp, polyester, spandex and so on. Compared with synthetic fiber, natural fibers have good moisture absorption and permeability, have the strength and stiffness of general fibers, small specific gravity, and better elasticity and extensibility. When used in protective textiles, it makes people comfortable to wear; the strength and toughness of synthetic fibers are generally better than natural fibers. Moreover, they are inexpensive and produced highly cost-effective. Although natural fibers and synthetic fibers have many advantages, they also have insurmountable defects, for example, cotton fabrics have poor elasticity, are easy to shrink and deform after washing, and lack of acid resistance; hemp fabrics have rough hand feelings, and they have burrs when worn close to the body, not smooth and comfortable, etc. The short-chopped fibers produced in the processing and forming of synthetic fibers do serious harm to the skin and respiratory system of the staff. Moreover, traditional cut-resistant fiber materials don’t have a good protective effect, which can’t meet the protection requirements of people’s production operation. In order to make natural fibers and synthetic fibers more competitive, it is imperative to modify them and increase the added value of their use (Li and Luo, 2010; Zhai and Guo, 2021). It is very important to carry out proper surface modification treatment for fibers with smooth and inert surfaces. After the treatment, the surface roughness of the fiber is increased, the active group is increased, and the binding ability with other materials is enhanced, which can effectively make up for the shortcomings of a single type of fiber such as low reactivity and insufficient mechanical properties. In the coated cut-resistant fabric, successful surface treatment can improve the mechanical properties of the coating, improve its anti-cutting ability, further avoid the occurrence of operator accidents, and generate corporate benefits.

### PPTA

PPTA, also known as aramid 1414, is the most representative of aromatic polyamide fibers. It was first developed by DuPont of the United States in 1971 under the brand name Kevlar. It is the first valuable high-function fiber that is still being produced and used (Guo, 2005). PPTA has excellent properties such as ultra-high strength, high modulus and light weight, in which the specific strength is 5/6 times that of steel, the modulus is 2/3 times that of steel wire and glass fiber, the toughness is two times that of steel wire, and the density is only about 1/5 of that of steel wire. It is a kind of high-performance fiber with excellent comprehensive properties, the largest output and the most widely used (Yue and Li, 2019). The application of PPTA is mainly concentrated in aerospace, military protective equipment, automotive rubber material reinforcement and other fields.

**FIGURE 1:**
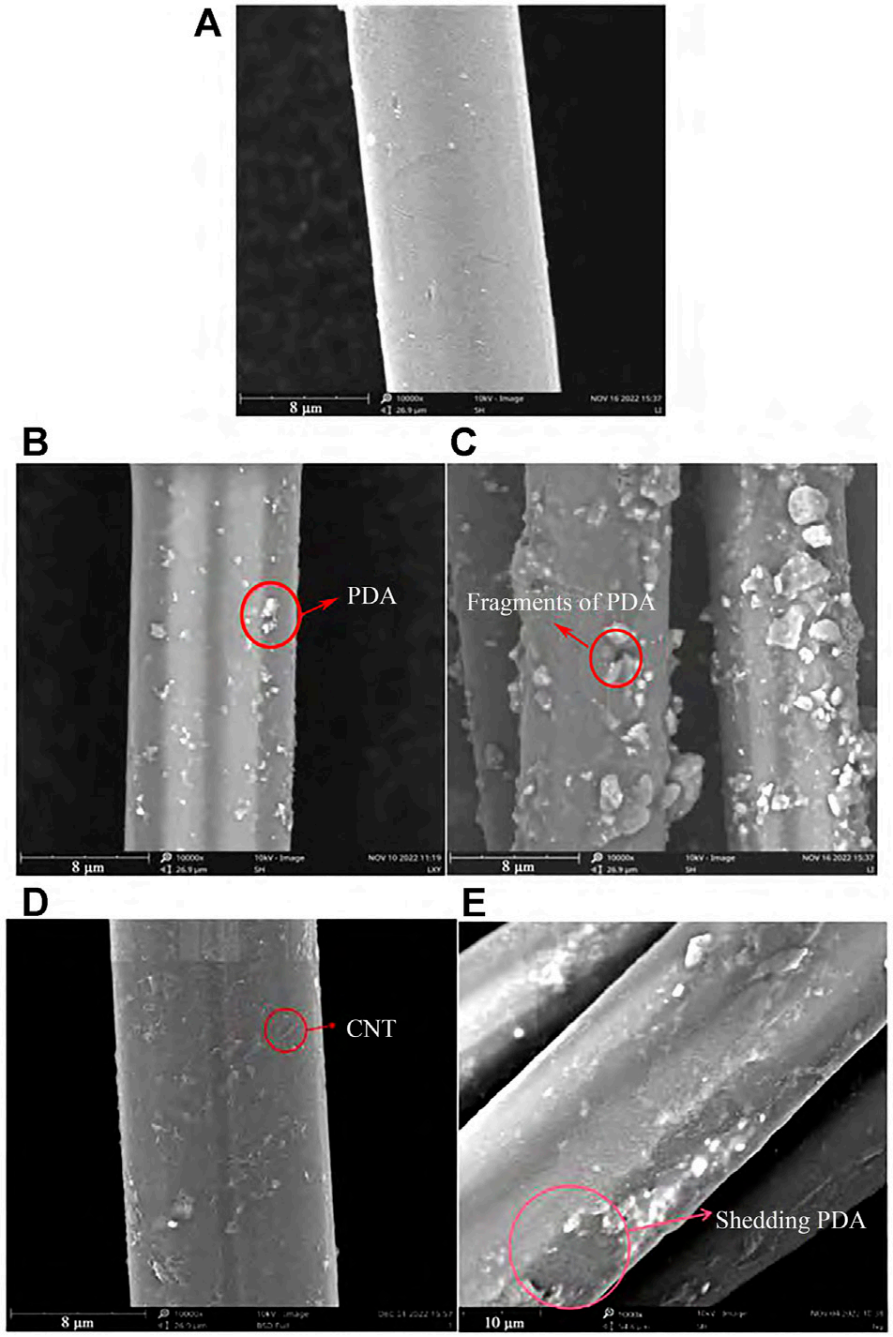
Surface morphology of aramid fiber before and after modification: **(A)** AF, **(B)** AF-PDA, **(C)** AF-PDA for 30 min, **(D)** AF-PDA-0.03% CNT, **(E)** AF-PDA-0.03% CNT for 30 min.

**FIGURE 2:**
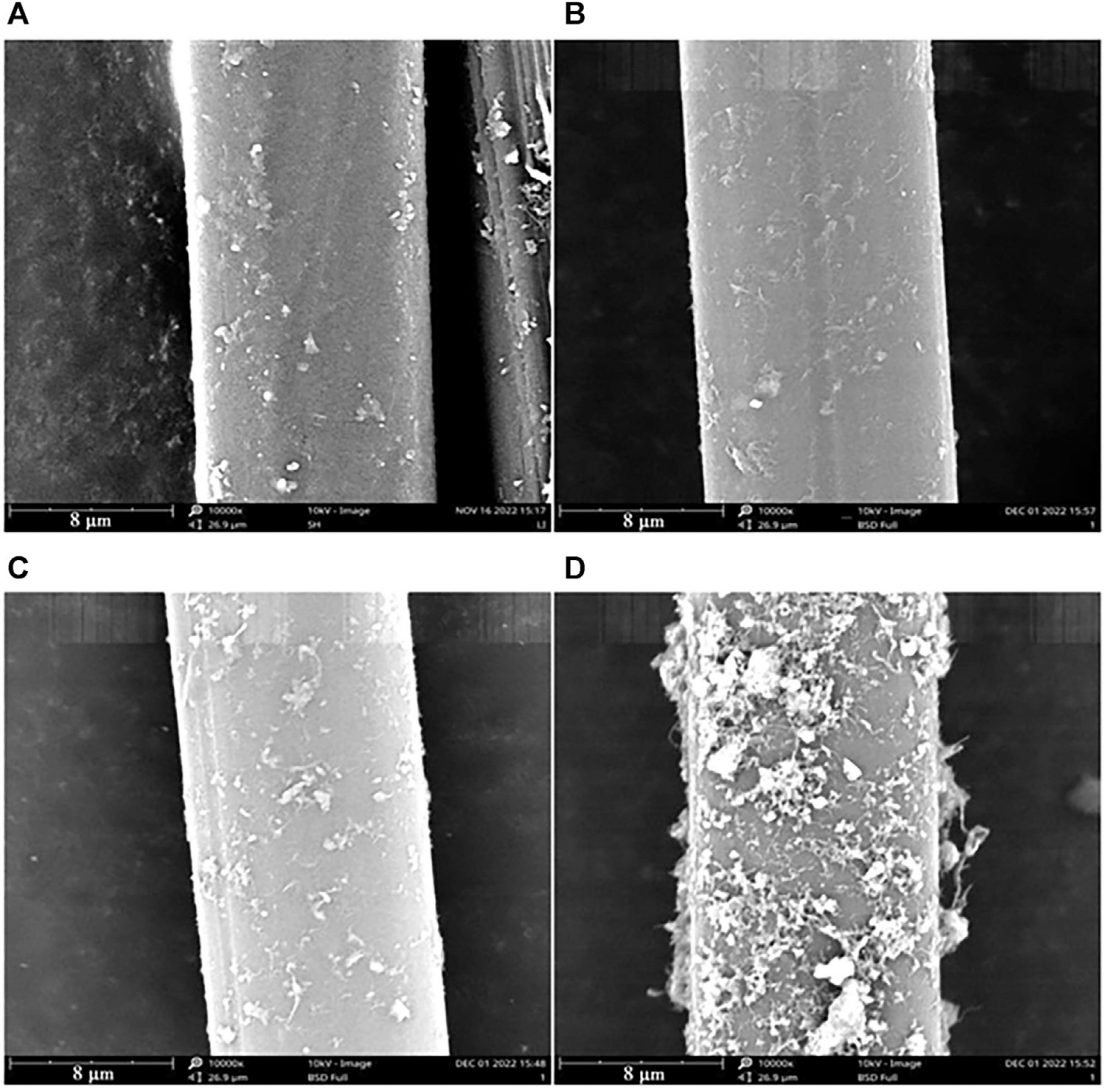
Surface morphology of modified aramid fiber with different concentrations of CNT added: **(A)** AF-PDA-0.01% CNT, **(B)** AF-PDA-0.03% CNT, **(C)** AF-PDA-0.05% CNT, **(D)** AF-PDA-0.1% CNT.

Based on the excellent performance of aramid, it is widely used in the field of protective equipment in China. Due to the uniform distribution of hydrogen bonds between molecular chains and the π-π stacking interaction of intermolecular benzene rings, this material has high strength and hardness, at the same time, the strong intermolecular interaction makes it difficult to dissolve in conventional organic solvents. And the molecular chain along the fiber axis highly oriented, the surface is smooth and inert, resulting in low reaction activity (Zhao and Gu, 2020). Researchers have conducted a lot of research and exploration on its surface modification. Common methods include surface chemical treatment and plasma treatment. Li and Li (2020), modified aramid fiber with dopamine-doped carbon nanotubes to improve the surface properties of aramid fiber and increase the interface strength between fiber and resin matrix. The SEM images of the surface morphology of aramid fibers treated with dopamine solution and dopamine-carbon nanotube solution are shown in Figures 1, 2, respectively. The results show that when the content of CNT is 0.03%, the carbon nanotubes on the surface of the modified aramid fiber are uniformly dispersed and the coating is densely covered in the fiber. Compared with the unmodified aramid fiber, the surface roughness of the modified fiber is improved, the active group is increased, the contact angle is decreased, the surface free energy is increased, and the binding force with the resin matrix in the composite material is improved.

**FIGURE 3:**
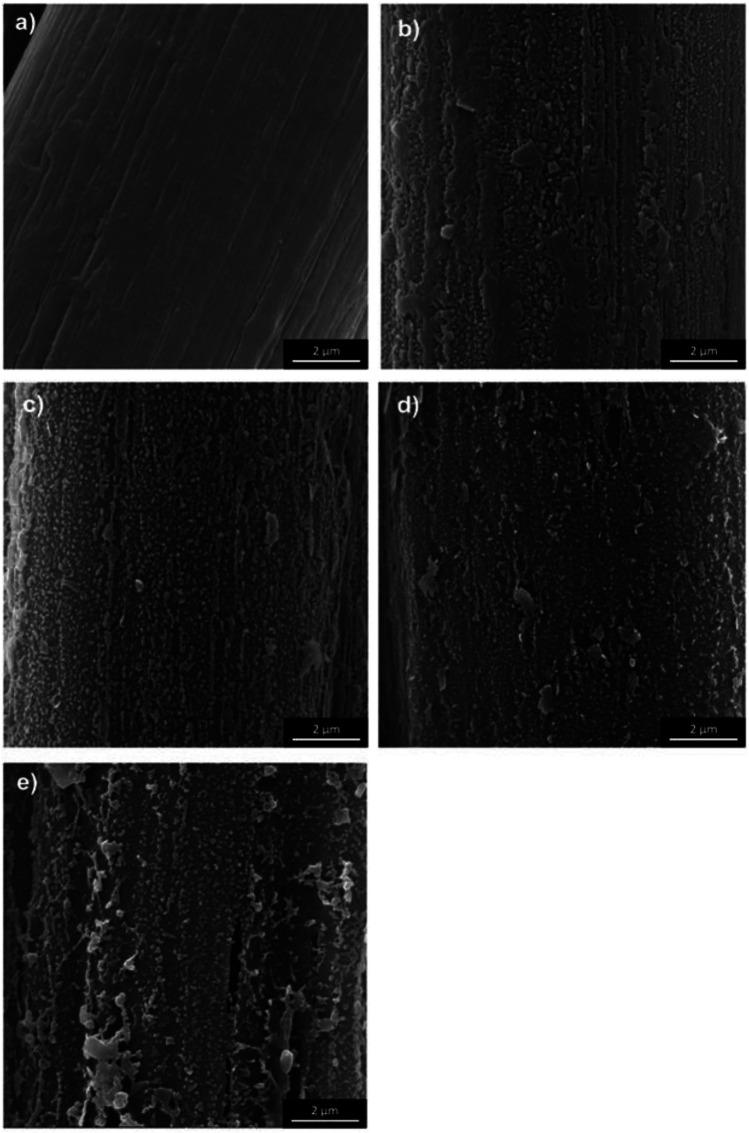
SEM images of the fiber surface of: **(A)** reference, **(B)** 10 min, **(C)** 30 min, **(D)** 60 min, **(E)** 90 min plasma-treated meta-aramid (mAr) yarn (magnification ×20,000).

 Zhaoqing Lu, Wenjing Hu et al. (Wu and Ma, 2021) used the sol-gel method to prepare the surface modification method of nano-SiO2 coated aramid fiber. The study found that a dense nano-SiO2 coating layer is formed on the surface of the modified aramid staple fiber, the friction and wear resistance of the fiber material are effectively improved. Zhao (2013) used phosphoric acid solution (H_3_PO_4_) to modify the surface of aramid fiber. The interfacial shear strength (IFSS) of aramid/epoxy composites material is increased significantly, but H_3_PO_4_ treatment has little effect on the mechanical properties of aramid fiber and can still meet the application requirements, which provides a new way for surface modification of aramid fibers. Nejman et al. (2020) treated meta-aramid and para-aramid yarns with low-pressure air radio frequency (RF) plasma for 10, 30, 60 and 90 min and observed the free surface energy, polarity and dispersion components. The morphology change of para-aramid treated by plasma is shown in Figure 3. The analysis shows that the surface morphology changes with the different plasma activation time, the unevenness and roughness increase. However, the mechanical properties of para-aramid fiber treated by low pressure air radio frequency plasma decreased slightly. Lv and Cui (2020) and others added untreated aramid staple fiber in the polyurethane synthesis process. It is found that with the increase of fiber content, the hardness, tensile strength and tear strength of the material gradually increased, and the elongation at break decreased, but within the experimental range, the fiber content had no significant effect on the wear resistance of the material. The development of surface modification technology has improved the composite fastness of aramid fiber and other materials to a certain extent, but it also has the defect of decreasing the properties of aramid fiber due to surface modification. Modification of aramid fiber by chemical treatment method takes a long time to dissolve, and there are problems such as difficult recovery of organic solvents, which easily pollutes the environment, and it is difficult to achieve large-scale production at present.

### Ultra High Molecular Weight Polyethylene

Ultra-high molecular weight polyethylene fiber is abbreviated as UHMWPE, which has excellent properties such as high strength, impact resistance, abrasion resistance, self-lubrication, chemical corrosion resistance, low temperature resistance and so on. It is an ideal material for artificial joint prosthesis, and is also widely used in aviation, medical transplantation, textile industry, paper making and many other fields. There are two production processes: one is the dry spinning process with decalin as solvent by DSM Company of the Netherlands, and the other is the wet spinning process with white oil as solvent by Honeywell Company of the United States. The fiber prepared by dry spinning has the characteristics of “fine denier and high strength, soft feeling, good spinnability and no solvent residue”, etc. The fiber is processed into cut-resistant gloves which have softer handle, better moisture absorption and perspiration effect and comfortable to wear (Wang and Zhang, 2020). The molecular weight of UHMWPE is more than 1 million, which is a kind of milky white powder with linear and high density, its structure is regular and easy to crystallize, the molecular chain in the crystal lattice is a plane zigzag shape, and there are no polar groups in the molecular chain, while its reaction activity is lower than that of PPTA. For this reason, people have carried out research work on fiber surface treatment, such as flame oxidation, chemical corrosion, low temperature plasma treatment and so on, in order to overcome this shortcoming of UHMWPE fiber.

**FIGURE 4:**
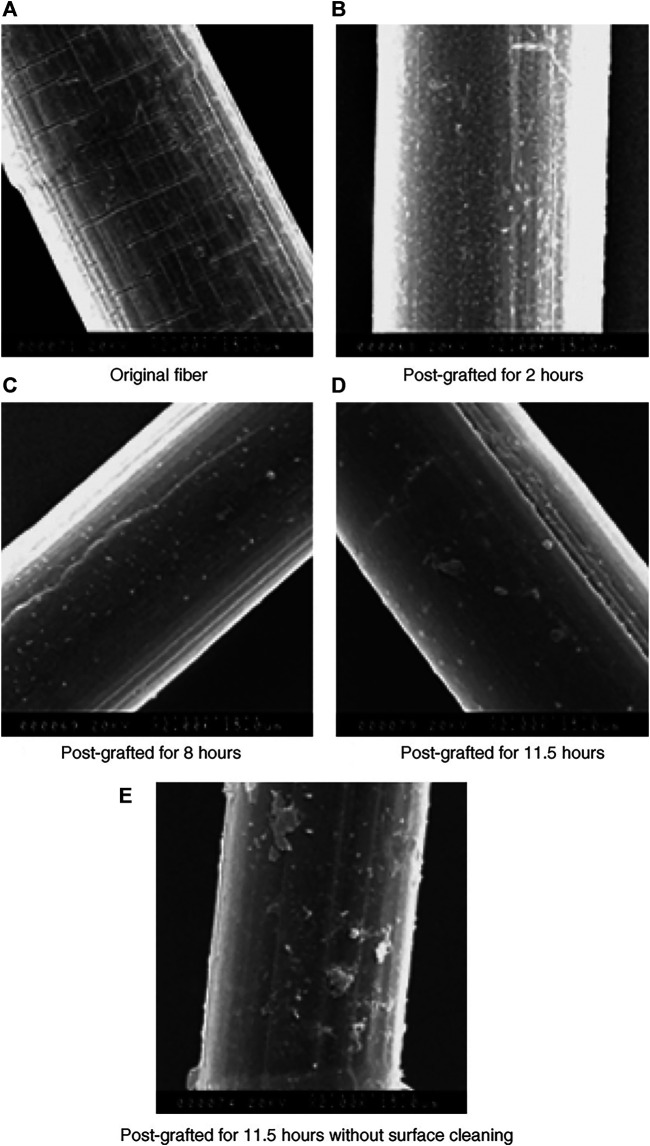
The SEM morphology of untreated and post-grafted DC88.

Piskarev et al. (Yang, 2015) modified the surface of UHMWPE with low temperature plasma. The results show that oxygen-containing functional groups are formed on the surface of UHMWPE film during the surface modification of UHMWPE film by oxygen plasma and air plasma, the adhesion of UHMWPE can be effectively improved. Zhao et al. (Piskarev and Gilman, 2017) successfully prepared a dense and uniform ZnO nanorod array on the surface of UHMWPE fiber by using a low-temperature hydrothermal method, which effectively enhances the interfacial bonding strength between the fiber and the resin, and this modification method had almost no effect on the intrinsic properties of the fiber, so it could be further used in industrial production. Wang et al. (Han and Shang, 2020) used high energy ultraviolet light to initiate the grafting reaction and grafted acrylamide onto the UHMWPE molecular chain, as shown in Figure 4. The study found that over time, the number of grafted products formed on the surface of the fiber increased, and the interfacial adhesion performance of the matrix was significantly improved. Technically, the disadvantages of UHMWPE of high inertness and low reactivity were effectively improved. Although the above three modification methods improve the bonding strength of UHMWPE and other composite materials to different degrees, they will all cause loss of fiber mechanical properties and environmental pollution, which is difficult to meet the requirements of enterprise production efficiency and current green and sustainable development. At present, the commonly used cut-resistant materials in China are mainly UHMWPE coated with spandex, glass fiber or steel wire, which meet the requirements of high wear resistance, cut-resistant and other properties required by protective clothing on the market.

Due to the better abrasion resistance and cutting resistance of UHMWPE, the Netherlands DSM Company began to develop UHMWPE cut-resistant gloves in 2000. At present, only four labor protection products manufacturers have been authorized to produce, and there are only two in China. In 2009, Beijing Tongyizhong developed 400D fiber for cut-resistant gloves, Hangzhou Xiangsheng and Yizheng chemical fiber also entered the market one after another, greatly reducing the production cost of cut-resistant gloves. Different from the rapid entry of foreign UHMWPE cut-resistant gloves enter into various labor industries, some domestic enterprises have insufficient knowledge of cut-resistant gloves, workers are not aware of their own prevention, and the promotion process of the use of cut-resistant gloves is relatively slow (Wang and Zhang, 2020). In recent years, due to the improvement of UHMWPE catalysis and polymerization technology, the product quality has been gradually adjustable and controllable; at the same time, the country has issued relevant laws to protect the personal safety of workers, production enterprises have increased demand for cut-resistant gloves, workers’ awareness of their own safety precautions has been improved, and the application fields and quantities have increased year by year. The domestic cut-resistant gloves industry is developing rapidly (Nejman and Kaminska, 2020).

### P-Benzoxazole Polyester Fiber

P-benzoxazole polyester fiber also is abbreviated as PBO, has the characteristics of high strength, high modulus, high temperature resistance and flame retardancy, with a strength of 5.8 GPA and a modulus of 280 GPA, which is another high-performance synthetic fiber after Kevlar fiber, and its strength exceeds Kevlar fiber and carbon fiber, etc., and it is by far the highest heat-resistant organic fiber. In 1964, the American Dupont Company first carried out the research, and was later developed by the United States Air Force Materials Research Office (So, 2000; Wang et al., 2006; Lu and Wang, 2018). In the spring of 1995, Japan’s Toyo Textile Company began trial production on the pilot plant of 1t/a under the patent license of Dow Chemical Company of the United States, and the 180t/a plant was officially put into production in October 1998 (Shi and Li, 2017). In 1993, Kitagawa et al. made a model of PBO based on the molecular structure of high-performance fiber proposed by Sawyer et al. PBO is composed of many fibrils and has an obvious skin-core structure (Wang, 1998). The PBO fiber cloth is mixed with shear thickening liquid to make the shear thickening liquid anti-stab cloth with high anti-stab performance, softness, comfort and good flexibility. It is used to make cut-resistant protective clothing, safety gloves, racing suits, etc., and has a good protective effect. In addition, PBO also has broad application prospects in aerospace, national defense, military industry, sports building materials and other fields because of its excellent performance.

**FIGURE 5:**
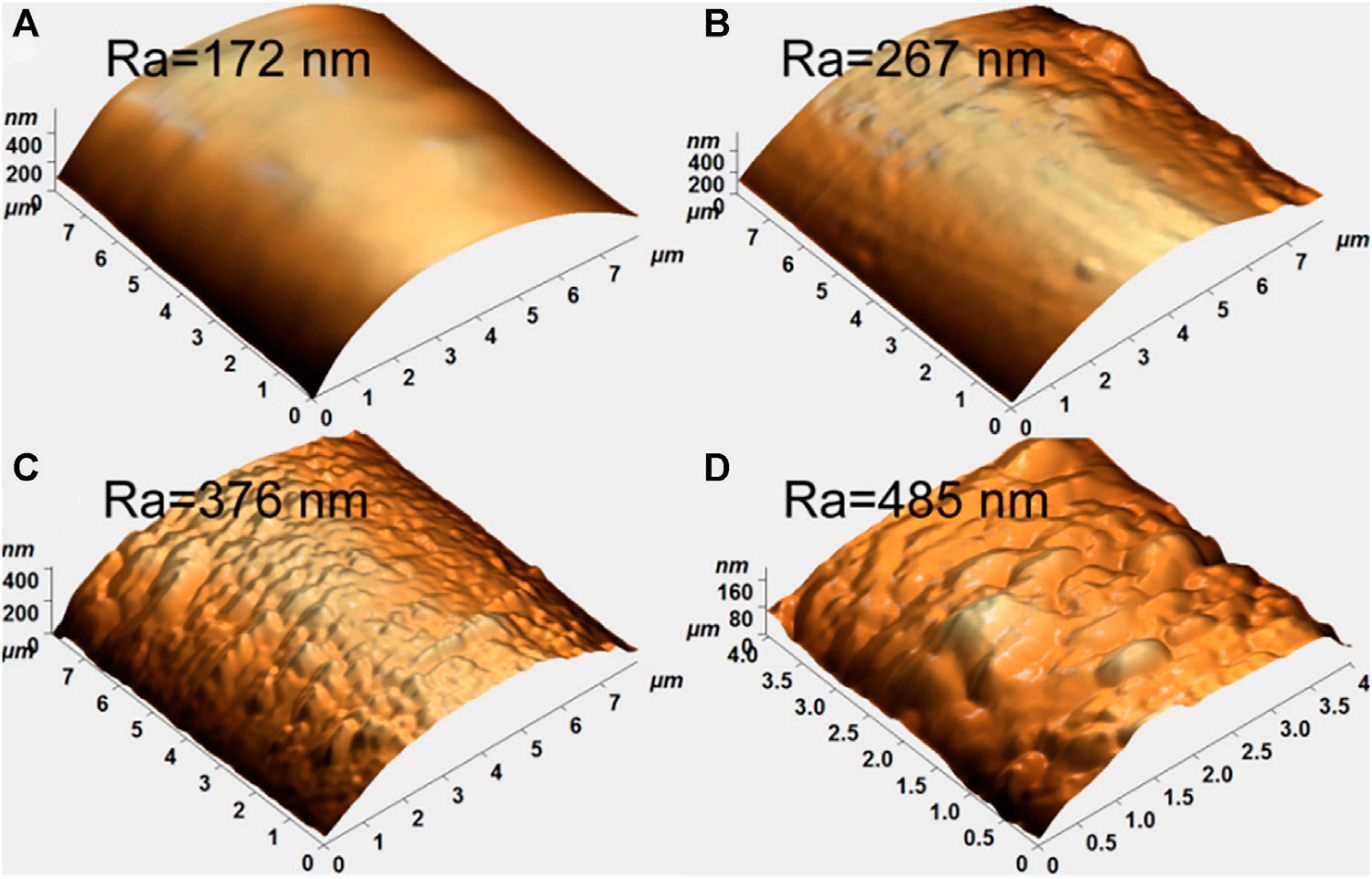
AFM images of untreated and treated PBO fibers.

The high anisotropy of PBO makes its axial compression performance far inferior to its tensile performance, and there is no large number of hydrogen bonds between molecular chains. It is easy to make the fibril unstable, resulting in kinks under pressure, which makes the fibers easy to crack and has low compressive strength. Because PBO fiber is a main chain liquid crystal polymer with extended chain conformation, straight and tight molecular chain, smooth surface and few polar groups (Ma and Ning, 2004), resulting in poor hygroscopicity, dyeing and adhesion with resin matrix. PBO fiber has poor light resistance, and exposure to light with a wavelength in the range of ultraviolet light to visible light will cause a decrease intensity, and the longer the exposure time is, the more the intensity decreases (Chen, 2018). At present, there are many methods to modify the surface of PBO fiber, and certain results have been achieved, but the excellent properties of the fibers have not been fully utilized. Song et al. (Dong and Xia, 2016) used oxygen ionomer induced vapor phase grafting to modify the surface of PBO fibers (Figure 5). The study found that the surface of untreated PBO fiber is smooth and striped. After oxygen plasma pretreatment with different time conditions, many protrusions and gaps are formed on the surface of the fiber, the surface roughness increases, and the bonding strength with the resin interface increases, but the tensile performance decreases, and the mechanical properties of the fiber have a certain loss. Liu (Song et al., 2012) observed the effect of argon plasma treatment on the interfacial bonding properties of PBO/BMI composites and it is found that the surface of the treated fiber is hydrophilic, but the surface modification effect decreases with the prolonged storage time, and the surface structure is unstable. In recent years, domestic and foreign researchers have done a lot of experimental explorations on the functional modification of PBO. Although the commonly used plasma modification method increases the surface roughness of the fiber and improves the bonding strength of the PBO fiber and other composite materials, as the treatment time increases, the surface roughness of the fiber continues to decrease, and the adhesion of the fiber surface decreases. With the deepening of research and the continuous maturity of production technology, PBO fiber, as a kind of high-performance fiber with excellent comprehensive properties, will continue to improve its modification methods. There is still much room for improvement in its application, and it will gradually cover various fields.

### Glass Fiber

**FIGURE 6:**
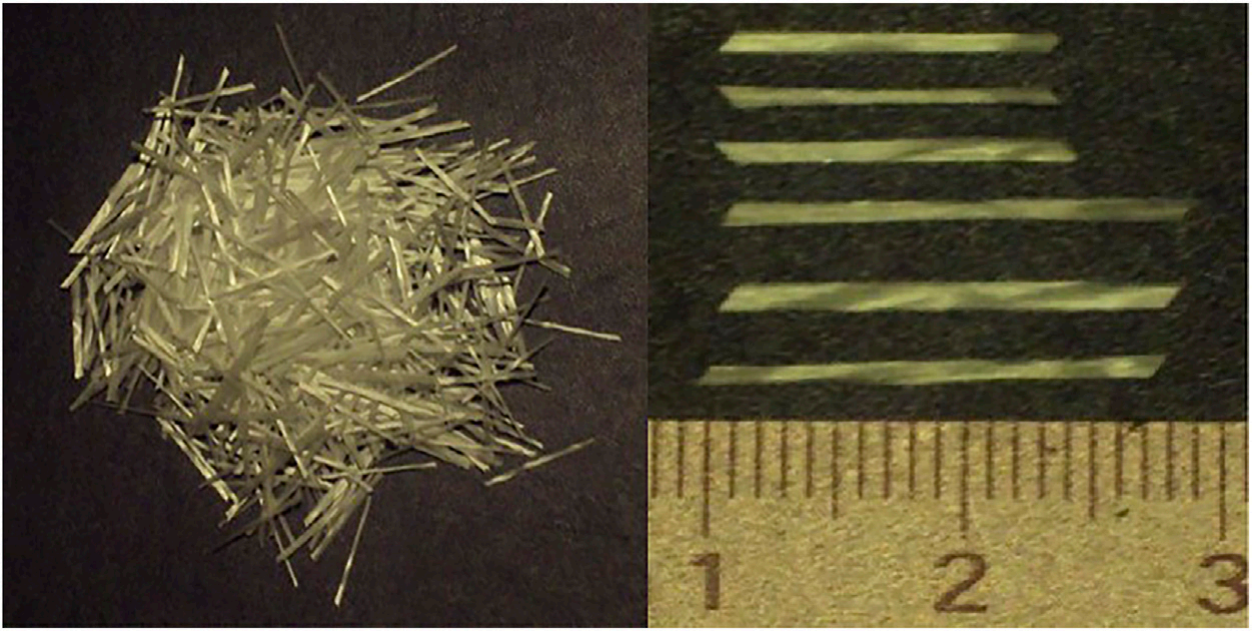
Glass fiber and geometry.

Glass fiber is a kind of inorganic non-metallic material, which is a fibrous material made of glass under the control of external force or thrown by centrifugal force in the molten state. The diameter of fiber monofilament ranges from a few microns to more than 20 microns (Figure 6), and it has excellent properties such as corrosion resistance, heat resistance, insulation and so on. Its strength far exceeds other natural, synthetic fibers and various alloy materials, and the tensile strength is 1,470∼4,800 MPa (Shi et al., 2021). Glass fiber as a kind of reinforced material can greatly improve the toughness, rigidity and durability of the composites (Liu et al., 2011). The composites are often compounded with aramid and UHMWPE to be used in protective textiles. Shi et al. (Wang and Chen, 2020) introduced BNN-30@BNNS filler into glass fiber reinforced epoxy laminate composites by hot pressing, which greatly improved the mechanical properties and interfacial shear strength of glass fiber composites. However, because the glass fiber is mostly short fiber, the protective clothing after composite weaving is easy to itch during wearing, and it may even cause allergic reaction in severe cases.

Glass fiber was produced in the late 1930s. Since the advent of glass fiber and the emergence of epoxy resin and unsaturated polyester, the era of composite materials composed of inorganic materials and organic materials immediately ushered in. From 1958 to 1959, the wire drawing process of glass fiber pool kiln created conditions for the large-scale and modern production of glass fiber. From the end of the 20th century to now, there has been a stable market for glass fiber reinforced thermosetting, thermosetting materials and used in electrical insulation and construction enterprises. China’s glass fiber manufacturing started in the 1950s. In recent years, China’s glass fiber output has gradually increased. In 2007, China’s glass fiber output accounted for 1/3 of the world’s total output, ranking first. With the in-depth study of the properties of glass fiber, the application of glass fiber in the industrial field has become more and more extensive (Tassew and Lubell, 2014).

### Metal Fiber

Metal fiber is a fiber material with a certain length-to-diameter ratio prepared by metal through a certain processing technology, which is different from the special fiber of traditional textile raw materials. Its fiber diameter can reach 1∼80 μm, which is thinner than that of hair (70∼100 μm). It has the advantages of good electrical conductivity, thermal conductivity, wear resistance, cut-resistance, high strength and high elastic modulus (Leong and Ramakrishna, 2000; Application of stainless steel metal fiber in new textile field Special Steel Technology, 2003; Liu and Zhang, 2009). Metal fiber was firstly produced by cluster drawing method in the United States in 1936, and was produced on a large scale by Bekaert Company of Belgium. At present, there are three main methods for the preparation of metal fiber: melt extraction method, drawing method and cutting method (Xu and Chen, 2019). Metal fiber not only has all the advantages of metal material itself, but also has some special properties of non-metallic fiber. There are two main kinds of metal fibers used in textile products: pure metal fiber fabrics and blended fabrics. Mainly used for anti-static, conductive, shielding, vacuuming fabric, etc. Because the metal fiber is soft and spinnable, it can be blended with other fibers to improve the cut-resistance and wear resistance of the material. In addition, metal fiber is also used in filter materials, anti-counterfeiting materials, fiber reinforced composites, etc.

**FIGURE 7:**
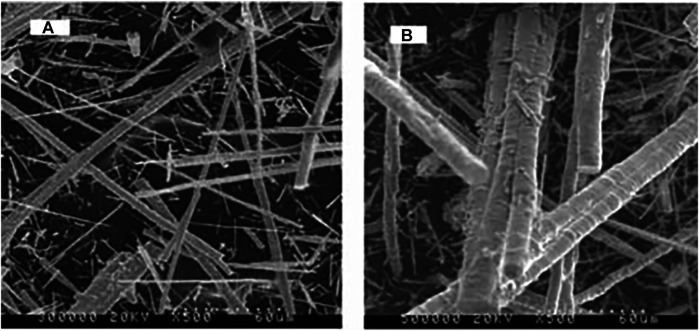
SEM images of the original **(A)** and SiO_2_-coated **(B)** magnetic metal fibers (×500).

With the expansion of the application demand of metal fiber, people put forward new requirements for its quality characteristics. New manufacturing methods such as dieless drawing, organogel-thermal reduction and electrospinning are constantly innovated and developed to meet the changing needs of the metal fiber market (Liu and Liu, 2005). In order to reduce the bending stiffness of metal wire yarns with high stiffness, high modulus and low extensibility to facilitate the knitting of knitted fabrics, Xu et al. (Ren and Liu, 2019) analyzed the effects of two different twisting processes on the surface morphology and bending stiffness of the yarns. It is found that under the same condition of the yarn linear density, the bending stiffness of the yarn can be reduced by reducing the linear density of the monofilament in the yarn, thus increasing the softness of the yarn and making it easier to weave. Xie et al. (2007) used the sol-gel method to modify the surface of the metal fiber, and coated the surface of the metal fiber with SiO_2_ (Figure 7). It is found that the surface of the metal fiber after SiO_2_ treatment is rougher than before, although the strength is slightly stronger However, this modification method greatly enhances the application range of metal fiber reinforced materials as protective materials. Aiming at the problem of the smooth surface of the metal fiber and low bonding strength with the matrix, Cho et al. (2013) used oxygen plasma, acid and alkali to modify the surface of metal fiber. The experimental results show that the surface of the modified metal fiber is rough and has good adhesion with aramid, polyester and other fibers, and has no great effect on its wear resistance and electrical conductivity. Metal fiber is widely used in civil and military protective materials and other fields because of its unique wear resistance, cut-resistance, thermal conductivity, etc. Metal fiber and its products have gradually developed from a single product to a wide variety of new materials, which not only provides high-quality protective products for the field of industrial protection, but also promotes the development of related industries in other fields.

The research and development of metal fiber in China began in 1979. However, due to the problems of technology and equipment, the quality of metal fibers such as stainless steel fibers produced in China is far from reaching the production level of similar products abroad. In the following 10 years, China’s metal fiber industry had gone through the process from importing raw materials to localizing raw materials and exporting finished products. In 1983, China successfully developed stainless steel fiber and established a number of metal fiber production bases, such as Northwest Nonferrous Metals Research Institute, Beijing Nonferrous Metals Research Institute and so on. As one of the new fiber materials in the high-tech field, metal fiber has made great progress in the world because of its excellent performance and promotes the development of other related fields (Xu and Chen, 2019).

## Coated Polymer for Cut-Resistant

Cultural relics were unearthed in the Jin Dynasty in China, and it was discovered that there are fabrics finished with *Dioscorea nigra*, which is an early coated fabric. Waterproof is the original purpose of coated fabric. With the application of modern technology, people have higher and higher requirements for the performance of coated fabrics (Zheng, 1986). It is required that the coated fabric should not only have protective properties, but also have breathability. Nowadays, the coated fabric has been widely used in the field of labor protection overalls, which puts forward higher requirements for coated fabrics, such as cut-resistance, flame retardant, stain resistance, wear resistance, etc. Coating is one of the key factors affecting the quality of cut-resistant textiles. The cut-resistant performance of the coated fabric is mainly achieved by improving the shear resistance of the coating material, increasing the friction of the coating material, and blocking the microporous structure of the fabric. As a qualified cut-resistant coating, the first characteristic must be extremely strong adhesion; high wear resistance, high strength and excellent flexibility are also reference factors for the selection of coatings.

At present, cut-resistant textile materials can be roughly divided into three types: 1) knitted uncoated textile materials; 2) knitted coated textile materials; 3) cutting and sewing textile materials. Among them, knitted coated textile materials are mainly used for cut-resistant vests, gloves, neck covers, etc., and are woven with cut-resistant yarns to form fabrics with certain protective properties, which are then immersed or partially immersed in polymer solutions with different compositions. It is made to improve its wear resistance, cut-resistance, static resistant and other properties. Despite the rapid development of modern coating technology, the basic methods of coating have not changed. The micropores of woven, knitted or non-woven fabrics are blocked by coating a layer of adhesive polymer film on the surface of the fabric (Xu, 1986). However, the cut-resistant coated fabric treated with anisotropic fillers have a slightly lower cut-resistant performance than the uncoated cut-resistant fabric. Because the anisotropic fillers block the pores of the fabric structure, when the knife cuts the fabric, the yarn cannot roll around itself and is difficult to slip along the cutting direction of the knife. Therefore the cutting resistance ability is reduced, and the durability of the coated fabric is further reduced which is not suitable for the workplaces that require high cutting capacity. However, due to its excellent comprehensive performance, cut-resistant coated fabrics are still widely used in the market. Commonly used cut-resistant coating polymers are generally divided into the following types.

### NBR

Nitrile rubber is an irregular high molecular polymer obtained by emulsion polymerization with butadiene and acrylonitrile as basic units. The content of acrylonitrile in NBR is one of the most important factors affecting the properties of vulcanizates. The content of acrylonitrile affects the ease of rotation in the rubber molecular chain and the intermolecular force, thus affecting the flexibility of the molecular chain and the physical and including mechanical properties and heat resistance. NBR has a wide variety. According to the content of acrylonitrile, it can be divided into ultra-high nitrile (42–53%), high nitrile (35–41%), medium high nitrile (28–34%), medium nitrile (24–27%), and low nitrile (16–23%) five types. From the perspective of application, it can be divided into ordinary type and special type (Xu, 1986). Compared with other rubber, NBR has a wider service temperature, can be used at 120°C for a long time, and has good low temperature resistance, with a glass transition temperature of −55°C.

**FIGURE 8:**
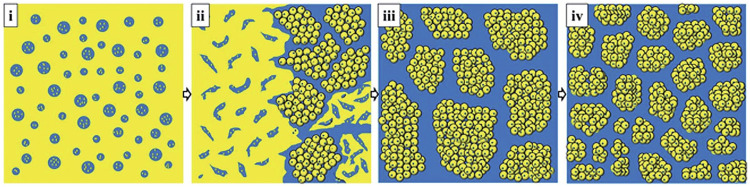
Schematic illustration of morphological evolution of NBR/PP TPVs during DV (the yellow regions represent PP phase and the blue regions represent the NBR phase).

The polar group nitrile group and unsaturated double bond in the molecular structure of NBR make it have good oil resistance and excellent physical and mechanical properties (Chen and Che, 2021). Anti-cutting coating materials with NBR coating generally have good chemical resistance and mechanical strength, wear resistance, puncture resistance, protein-free allergen, and the lowest allergic irritation to human skin (Xu and Wu, 2020). The anti-cutting coating material made of nitrile-butadiene rubber provides better protection than the natural rubber coating (Espasandín-Arias and Goossens, 2014). However, there are a large number of unsaturated double bonds in the molecular structure of NBR, which are prone to cross-linking and aging under heat or thermal oxygen conditions. In order to delay aging and increase the service life of NBR, antioxidants are generally added in the production process. With the breakthrough of production technology and process, people have put forward higher requirements for the performance and environmental protection of NBR. Ning et al. (2017) used dynamic vulcanization to prepare oil-resistant nitrile rubber (NBR)/polypropylene (PP) thermoplastic vulcanizate (TPV). A schematic diagram of the morphological evolution of NBR/PP/TPV during vulcanization can be seen in Figure 8. It is found that although dynamic vulcanization increases the density of NBR polymers and the crystallinity of NBR/PP/PVC blends, the mechanical properties, elasticity and oil resistance of NBR/PP/PVC blends are improved, the rheological properties decreased significantly. Which increased the uniformity of the cut-resistant coating textile material dipping difficulty, resulting in a certain limitation of its application range.

### Rubber

Rubber is divided into natural rubber (NR) and synthetic rubber (SR). Generally speaking, natural rubber refers to natural rubber latex collected from rubber trees, rubber grass and other plants, with various ingredients as auxiliary materials, and elastic solids made by processes such as rubber mixing, calendering, extrusion, molding, and vulcanization. Natural rubber has the advantages of good electrical insulation, abrasion resistant, good biodegradability and so on, so it is a kind of renewable natural resources with superior comprehensive properties. In a broad sense, synthetic rubber is a highly elastic polymer synthesized by chemical methods. In recent years, the synthetic rubber industry has developed rapidly and the production scale has been continued to expand. Since natural rubber is non-polar rubber and contains unsaturated double bonds in the molecule, its thermal and oxidative aging resistance, ozone resistance and UV resistance are poor (Yao and Huang, 1994; Wang and Zhang, 2008; Zheng and Zhong, 2014). These defects have gradually restricted the application of natural rubber. In order to make better use of this natural renewable resource, people have devoted themselves to the modification of natural rubber for a long history.

The essence of the modification of natural rubber is the change of structure, including the change of chemical structure of molecular chain and changes in the aggregation state. Modification methods are mainly divided into chemical modification and blending modification. Among them, chemical modification mainly uses unsaturated double bonds in natural rubber to carry out various chemical reactions and introduce new functional groups to improve the air permeability and flame retardancy of rubber products. The blending modification is to introduce other components into the rubber matrix so as to achieve the mechanical properties of the rubber coating material. Gu and Li (2009) used sulfuric acid to acid hydrolyze natural microcrystalline cellulose (microcrystalline cellulose, referred to as MCC) to prepare nano-microcrystalline cellulose whiskers, then added natural rubber latex, after coprecipitation, kneading and vulcanizing, got the NR/RH composites, and then modified by adding resorcinol (R) and hexamethylenetetramine (H) to obtain NR/RH-CW composites. It can be seen from Table 1 that the addition of CW improved the mechanical properties of NR, and the research results show that CE has a significant reinforcing effect on natural rubber. Xie and Zhang (1997) summarized the chlorination processes of several natural rubber and synthetic rubber, analyzed the structure and reaction mechanism of chlorinated rubber. However, because CCl4 is toxic and is recognized as an atmospheric ozone depletion agent, developing a new technology for producing chlorinated rubber with non-carbon tetrachloride solvent to meet the products demand is one of the important trends in the development of natural rubber modification technology in recent years. Moreover, since the most of the auxiliary raw materials of rubber products except natural rubber are powder, particles and organic waste gas will be produced in rubber refining, vulcanization and other production processes, resulting in environmental pollution (Zhang and Qi, 2019).

**TABLE 1:** Mechanical properties of 1NR/CW composites.

Sample (phr)	Modulus at 300% (Mpa)	Tensile Strength (Mpa)	Elongation at break (%)	Tear strength (k N/m)	Pemaneat set (%)	Shore A hardness
NR	2.7	22.0	579	29.3	20	43
NR/CW(100/3)	2.9	25.9	564	32.6	20	46
NR/CW(100/5)	2.9	28.1	576	31.7	24	45
NR/CW(100/8)	2.9	28.7	578	31.1	22	46
NR/CW(100/10)	3.0	27.1	612	31.8	24	48
NR/CW(100/12)	3.5	27.6	627	31.1	34	49
NR/CW(100/15)	3.9	26.8	604	35.0	32	50

**FIGURE 9:**
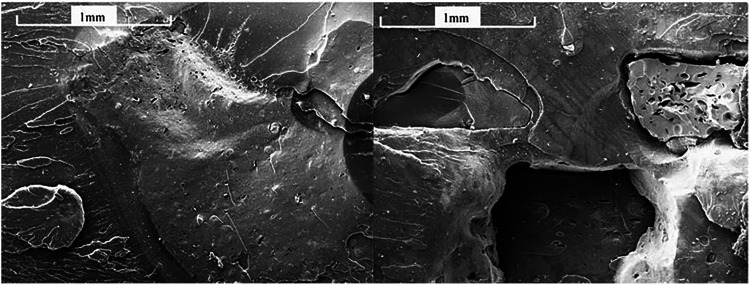
SEM of Dev. R 10 phr tensile fractured surface **(A)** and Dev. F 10 phr tensile fractured surface **(B)**.

In order to maintain sustainable development and meet the requirements of green chemistry, many scientists are committed to finding natural materials to replace synthetic materials completely or partially, thereby recycling or reducing the use of plastic and rubber products. Lamminmäki et al. (2006) used continuous shear flow level control reaction technology to selectively break the sulfur cross-linking in rubber and continuously produce high quality vulcanized rubber. The desulfurized rubber produced from scrapped tires was used as the rubber component of filler (Dev. F) and natural rubber (NR) (Dev. R), and the properties of the obtained rubber compounds were studied and analyzed. By observing the electron microscope of the tensile fracture surface (Figure 9), it was found that adding 15 phr of Dev. F, or even up to 50 phr of Dev. R had no obvious effect on the mechanical properties of natural rubber. Even when the Dev. R compound contains 10 phr of waste rubber, its tensile strength and elongation at break were higher than those containing raw rubber. This provides a new technical way to develop the use of waste rubber and reduce the production cost. Masłowski et al. (2021) used nettle as natural fiber filler to modify natural rubber. The experimental results show that adding nettle to the natural rubber composite material can enhance the mechanical properties of natural rubber, and it has been widely used in the coating process of bullet-proof and cut-resistant textile materials. The preparation of composite rubber materials with natural fiber fillers reduces the production of toxic by-products, develops the potential application of natural fiber fillers, and provides a new solution to solve the environmental pollution in the process of rubber production.

### PVC

PVC actually is a kind of vinyl polymer, which is a non-crystalline material with excellent properties such as non-toxic, tasteless acid and alkali resistance, oil resistance, good water resistance. Because of its non-allergic reaction to human body, Economical and good hand feeling, it is widely used in all kinds of protective fabric coating, especially as cut-resistant coating polymer coated on the surface of high-performance fiber to achieve enhance cut-resistance, waterproof and insulation of cut-resistant textiles. With the progress of chemical technology and the gradual improvement of industrial supporting facilities, as well as the continuous application of new auxiliaries, the large-scale production process of PVC coated fabrics, especially PVC gloves, is becoming increasingly mature. At present, China is the largest producer of PVC coating products, Almost all the products are exported, while the domestic market accounts for a relatively low proportion of the total production. With the continuous development of China’s economy and the stimulation of special events such as new type of coronary pneumonia, people’s awareness of self-safety protection has gradually improved and consumption concepts and habits are also constantly changing, the domestic application of PVC coating products will become inevitable (Feng, 2017).

**FIGURE 10:**
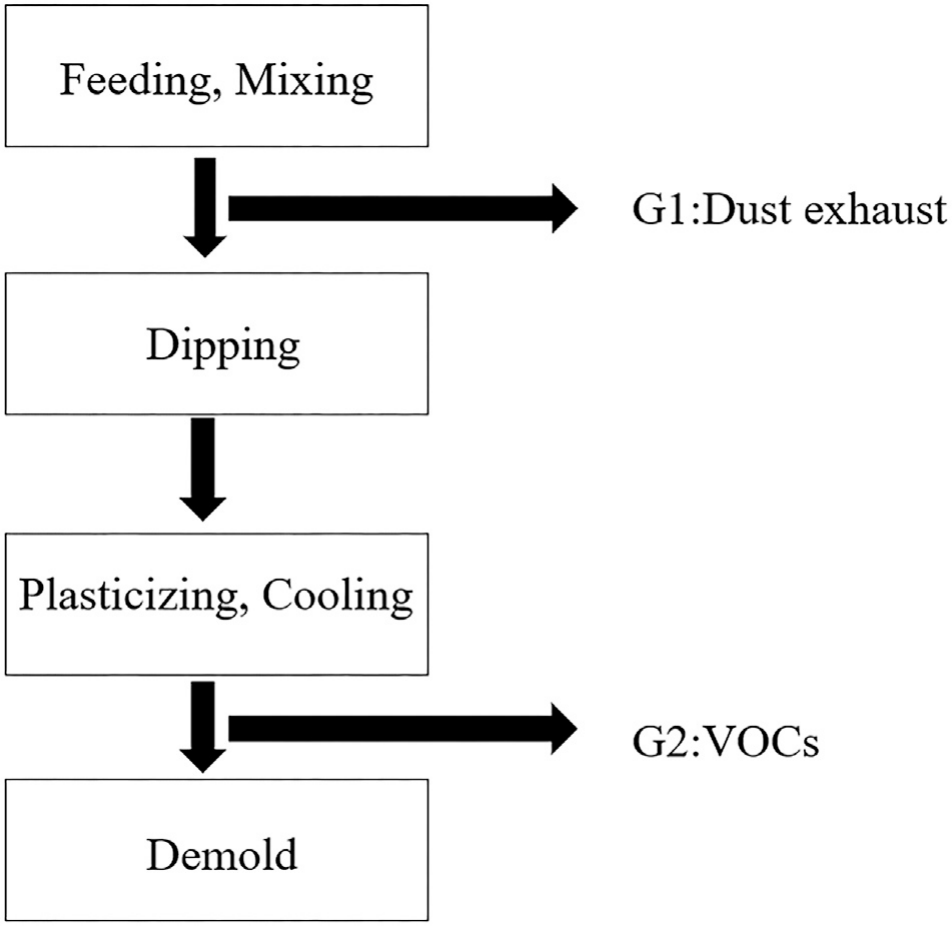
PVC glove production process.

PVC coated fabrics are highly recognized by consumers due to its good protective properties and excellent cost performance. However, the surface of PVC coated fabric is astringent and difficult to wear. It will become brittle when used in a low temperature for a long time, which not only destroys the aesthetics of the product, but also affects the protective performance of coated fabric (Li and Yang, 2009). In order to meet the needs of people, other additives such as plasticizers, lubricants and auxiliary processing agents are often added in the production process. Taking PVC gloves as an example, the production process is shown in Figure 10. It is not difficult to find that in the production process of PVC gloves, auxiliary processing agents such as plasticizers and lubricants will generate a large amount of dust, exhaust gas and volatile organic compounds, which not only pollute the surrounding environment, but also seriously endanger people’s health. Among them, phthalate plasticizer (DOP) is the main plasticizer commonly used. As early as a few years ago, the European Union explicitly listed it as a prohibited item in REACH and other regulation. At present, in response to the national green production requirements, some manufacturers have begun to develop alternative materials according to the market demand. Jun Zeng et al. (Liu, 2020) used the acetone method to prepare PVC glove coatings for the difficulty of wearing PVC protective gloves. The study found that the low temperature stability of the synthetic latex coatings is improved, the tensile strength of the film is better, and the surface of the gloves is smoother. Xie (2017) from the perspective of poor low temperature resistance of PVC coated fabrics, explored the methods of modifying the low temperature resistance of PVC with several kinds of nano-rubber particles and found that the addition of NR-541 rubber particles could greatly improve the low temperature resistance of PVC. Le (Ying, 2009) aimed at the productions of harmful substances in PVC coated fabrics, outlined several PVC welding technologies, analyzed the feasibility of TPU and EVA as substitute materials, and pointed out that alternative materials should be modified to improve them. Weldability is the development direction of PVC substitute materials in the future. Because PVC contains a large amount of chlorine, the chlorine content is about 57%. In order to avoid secondary pollution to the environment, PVC must be recycled reasonably. YH, Lu et al. (Lu et al., 2021) used isothermal thermogravimetric method to study the thermal degradation process and isothermal discoloration of PVC in air and 453–503 K, and used chemical kinetic method to fit the single equation model of mass loss and time in the process of thermal oxidative degradation of PVC. It was found that the calculated results were basically consistent with the experimental data. In order to recover PVC, and prevent its carbonization, the thermal oxidative degradation time of PVC should be less than 130 min and at a temperature of 486k.

In addition, there is a common problem that the tear strength of PVC coated fabrics decreases too high, and some tear strength losses are as high as more than 50%. Deng and Yuan (2002) chose dioctyl phthalate (DOP) and dioctyl adipate (DOA) as main plasticizer, chlorinated paraffin (PCL) as radiation plasticizer to form the plasticizer system. PVC coated fabrics are tested for tear strength and fabric flexibility. The experimental results show that the tear strength of coated fabric can be decreased with the increase of total plasticizer content or the decrease of radiation plasticizer content. In recent years, with the advancement of science and technology, people have higher and higher requirements for the performance of PVC products. Because of the defects such as environmental pollution and poor low temperature resistance of PVC coating materials during the production process, some researchers are committed to looking for new types alternative materials to PVC. At present, PVC coated fabrics have been gradually replaced by PU resins with good properties and convenient processing.

### PU

Polyurethane is mainly produced by the reaction of polyols and isocyanates to form -NHCOO- unit. In addition to its structure containing urethane bonds, it also contains unsaturated bonds such as ester bonds, ether bonds, and urea bonds, which not only has the properties of amide group, such as strength, abrasion resistance, oil resistance, but also has the heat resistance and solvent resistance of polyester, as well as the water resistance and flexibility of polyether. It is widely used in military and national protection and other fields (Xu and Zhao, 2016; Feng, 2021). Polyurethane was first synthesized by Professor Bayer in Germany in 1937. Several commonly used polyurethane materials include: polyurethane soft foam, polyurethane rigid foam, polyurethane semi-rigid foam, polyurethane elastomer, polyurethane slurry, polyurethane coating, polyurethane adhesive and polyurethane sealant, etc. With the continuous upgrading and development of modern industrial production technology, polyurethane coating as a new type of coating, developed rapidly in the second half of the 20th century. Coating polyurethane coating on cut-resistant textiles can enhance the aesthetics, wear resistance, flexibility and impact resistance of cut-proof textiles, and have a good shock absorption and cushioning effect. In recent years, the polyurethane industry has made great progress in China, and China has become the fastest growing market center of polyurethane in the world. Yantai Wanhua Polyurethane Co., Ltd. has solved the key technical problems and successfully developed MDI production technology through independent scientific research and developed cooperation with scientific research institutions. At present, Yantai Wanhua Polyurethane Co., Ltd. has become the fifth enterprise in the world with independent property rights of MDI. In addition, China’s technological progress in production, application and other fields has also advanced by leaps and bounds. Compared with PVC coating materials, polyurethane coating materials not only have outstanding properties in wear resistance and elastic memory, but also have better low temperature resistance than PVC materials. While the production and recycling process of PVC coating materials will cause a lot of pollution to the environment. Waterborne coatings are widely used in polyurethane coating materials so that polyurethane coating materials can not only provide convenient conditions for the construction environment, but also help to prevent environmental pollution problems (Weng, 2008; Zhu and Weng, 2012).

**FIGURE 11:**
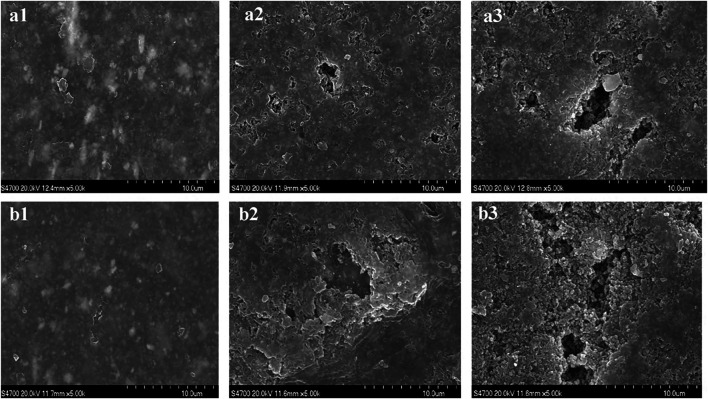
Surface morphology of the polyurethane composite coatings immersed in 3.5% NaCl solution for 3 h, 2.25 and 35 days, respectively: **(A)** acrylic polyurethane composite coating and **(B)** aliphatic urethane composite coating.

**FIGURE 12:**
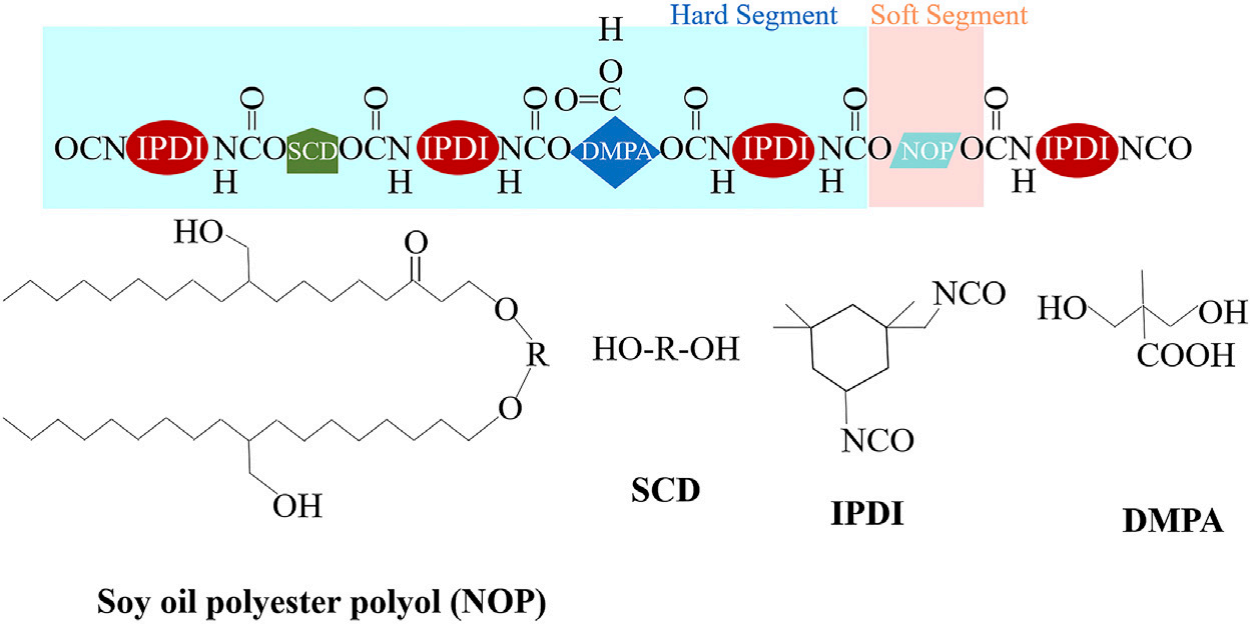
Schematic illustration of structure of NOP-PUDs.

Polyurethane is widely used in aerospace, civil protection and other fields due to its excellent comprehensive properties. However, with the increasing awareness of environmental protection and the continuous upgrading of industrial technology, people put forward higher requirements for its mechanical properties and environmental protection. In addition, polyurethane coating materials are susceptible to the effects of ultraviolet rays, temperature, water and chemical media, which reduces their durability, which greatly affects the reliability, safety and storage life of cut-resistant textile materials (He and Deng, 2002). In view of the shortcomings of various properties of polyurethane products, researchers are committed to modifying polyurethane materials to improve their durability, heat resistance and mechanical properties, looking for alternative environment-friendly auxiliary additives, and developing environment-friendly polyurethane products. Zhu et al. (2010) studied the UV radiation resistance of acrylic polyurethane composite coating and aliphatic polyurethane composite coating in 3.5% NaCl solution, and analyzed the damage mechanism of the coating. By observing the changes on the surface morphology of polyurethane composite coatings under different immersion time (Figure 11), it is found that rough pores appear on the surfaces of the two coatings, but the C-O bond of aliphatic polyurethane break faster and the coating is more damaged. Seriously, the acrylic polyurethane composite coating can provide better protection. Lyu et al. (2018) summarized several modification methods of polyurethane coatings, such as nano-particle modification, silicone modification and vegetable oil modification, and pointed out that the properties of polyurethane coatings were greatly improved and the application range of polyurethane coatings had been expanded at the same time. Oh et al. (2011) used a high-throughput method to explore the feasibility of modifying polyurethane dispersions (PUDs) by natural oil polyester polyols (NOPs) in order to develop environment-friendly adhesives with volatility. The structure of NOPs-PUDs is shown in Figure 12. The experimental results show that the addition of NOPs improves the water resistance, acid and alkali resistance and impact resistance of the final NOPs-PUDs polymer. The results of NOPs modification experiments on PUDs provide an effective basis for the selection of adhesives for high-performance polyurethane coatings in the future, and effectively promote the steady progress of green production of polyurethane coatings. Xia and Zhu (2017) used fluoroacrylate monomers and multifunctional mercaptans to blend and modify polyurethane, and their study found that the durability, yellowing resistance and elongation at break of the modified polyurethane coating were significantly improved. However, the current research on polyurethane modification technology cannot fully meet people’s performance requirements for polyurethane coating materials. Polyurethane coating materials generally have the problem of photodegradation after being irradiated by ultraviolet rays, which leads to a decrease in coating performance, and it is still necessary to further improve the polyurethane coating material. The mechanical properties of the coating continuously improve the coating process. Vigorously develop water-based polyurethane coatings, focusing on the development of polyurethane coatings with multiple protective functions and better environmental performance.

## Evaluation Standard of Anti-Cutting

According to different aspects of protection, there are many standards for functional textiles. This paper mainly discusses the performance of textiles, and its test standard mainly refers to the test standard of anti-cutting performance of cut-resistant gloves, so several standards related to cut-resistant gloves are introduced. At present, several authoritative cutting prevention and evaluation standards in the world mainly include Chinese National Standard EN420, European standard EN388, American standard ASTMF-1790 and international standard ISO13997.

### EN420 Standard

The EN 420 standard provides general indicators for the production and sales of protective gloves, including glove size, marking, flexibility, mechanical risk prevention standards, etc. The purpose of this standard is to ensure that the gloves themselves do not cause harm to the wearer and is comfortable to wear. At present, it has been adopted by the national standard of China (Fang and Tian, 2006).

### EN388 Standard

The EN 388 standard was adopted by the European Standards Committee (CEN) on March 16, 1994. It is suitable for protection against physical and mechanical injuries caused by friction, knife cutting, stabbing, tearing, chopping, etc., and is not suitable for shockproof gloves. The cutting principle of this standard is that the round blade under certain pressure (5 N edge pressure) performs a reciprocating movement within the specified distance range (50 mm) of the material, and records the number of cycles of the circular blade rotation at the same time, which is converted into the anti-cutting performance index after comparison with the standard material. Each sample is tested five times, and the sharpness of the blade is tested before and after each test to avoid inaccurate test results due to the different sharpness of the blade. When clamping the sample, place a piece of aluminum foil on the rubber pad to fix it, and the sample cannot be stretched.

When the standard sample meets the sharpness requirement for five times, record the number of operating cycles of the blade C_n_ (*n* = 1–5). Cut the test sample five times, record the number of blade rotation cycles T_n_ (*n* = 1–5), when the cut through occurs, then cut the standard sample five times, and record the number of blade rotation cycles C_n+1_ (*n* = 1–5). Calculate the anti-cutting performance index I. Where ‾C_n_= (C_n_ + C_n+1_)/2, I_n_ = (‾C_n_ + T_n_)/C_n_, the anti-cutting performance index $I=\overset{5}{\underset{\text{n}=1}{\sum }}\text{In}$ , the test method used in this standard is also applicable to arm protection equipment independent of gloves and clothing.

### ASTM F-1790 Standard

The ASTM F-1790 standard was approved by the American Society for Testing and Materials (ASTM) on June 10, 1997. It is the standard for testing the anti-cutting performance of materials, and the cutting is limited to the surface of the material. The cutting principle is that the ability of the material to resist the cutting of the cutting tool is determined by the distance crossed by the sharp cutting tool. The blade cuts through a certain distance from the surface of the material, and the final characteristic value is the cutting force, which distinguishes the material performance according to the size of the cutting force.

The sample needs to be insulated when it is fixed and cannot be stretched. The screw drive system makes the sample holder and the cutting knife move relative to each other, and the cutting edge is 90 ± 2 with the long axis of the sample fixture, and the relative motion speed is 2.5 ± 0.5 mm/s. The blade clamp should be able to firmly clamp the blade and make the blade exposed width of 12.0 + 0.5 mm. The measurement distance is from the static position of the blade in contact with the sample to the point where the sample is cut, accurate to 0.1 mm. The batch of blades used in the test should be not less than 200, the thickness of chloroprene rubber (1.57 ± 0.05 mm) mm, HRC (50 ± 5), the force applied on the blade on time is 5 mm 0.02 N, the average cutting length is 20–30 mm, the offset of the calibration factor of the measured blade should be less than 10%, and the calibration factor of the blade:

$$C=\frac{20}{\text{I}}$$

I is the cutting length on the rubber with the blade 5 N is applied. Test at least 15 numbers for the same set of samples, which are five data points within the following three cutting distances (3∼15 mm, 15∼30 mm and 30∼50 mm), and then plot the data into coordinate diagrams, the force required to extrapolate 20 mm is the final data of the material tested according to this standard (one blade must be changed each time).

### ISO 13997 Standard

ISO13997 standard is a standard on protective clothing issued and implemented by the International Organization for Standardization on August 15, 1999. This standard is consistent with the principle of ASTM F-1790, except that some parameters are slightly different. The standard clearly defines the scope, sample preparation, test equipment and test methods.

By comparing three different anti-cutting evaluation standards, it is not difficult to see that although EN 388, ISO 13997 and ASTM F-1790 adopt different testing methods, they all use standard reference samples for comparative analysis. Differences in measurement results caused by different sharpness. The quality of the standard reference sample will directly affect the test result of the sample material. Standard samples should be tested before sample testing to meet the requirements specified by the standard. The cut-resistant performance test standard comparison is shown in Table 2.

**TABLE 2:** Comparison of three anti-cutting evaluation standards.

Standards		EN 388 index	ASTM F-1790 (g)	ISO 13997 (N)
Evaluation scope		Wear, blade cutting, piercing and tearing	Blade cutting	Blade cutting
Blade shape		Round blade	Straight blade	Straight blade
Blade selection		Made of tungsten steel	Made of stainless steel	Made of stainless steel
		HV740-800	HRC>45	HRC>45
		Diameter 45 mm	Blade length 65 mm	Blade length 65 mm
		Thickness 0.3 mm	Width>18 mm	Width>18 mm
Cutting angle		30°–35°	22°	22°
Cutting speed		100 mm/s	2.5 ± 0.5 mm/s	2.5 ± 0.5 mm/s
Cutting motion		Horizontal reciprocating movement of blade	Relative motion between sample fixture and cutter	Relative motion between sample fixture and cutter
Performance grade	Level 1	1.2	<200	-
	Level 2	2.5	>200	-
	Level 3	5.0	>500	-
	Level 4	10.0	>1,000	>13 N
	Level 5	20.0	>1,500	<22 N

In terms of the scope of testing, EN388 can not only test the cut-resistant performance of protective gloves, but also applies to the forearm protective equipment of clothing, while the American standard ASTM F-1790 and international standard ISO 13997 are only suitable for material testing of protective clothing. In addition, EN 388 also uses different methods to test the friction resistance, tear resistance, and puncture resistance of materials, and classifies them according to the test results, which are represented by different mechanical hazard symbols. In the choice of knives, unlike the round blades made of steel and tungsten used in the European standard EN 388, the American standard ASTM F-1790 and the international standard ISO 13997 use the same standard straight blades made of stainless steel. The round blade and the straight blade adopt different hardness standards. The round blade reciprocates horizontally on the sample, and the straight blade moves relative to the sample holder. The two blades use different cutting angles and cutting speeds. The experimental test method itself also has certain problems. EN 388 does not specify certain experimental parameters. The cutting direction of the blade has a great influence on the results of the experimental test. The screw tightness on the sample carrier and the blade lubricant will also have a certain effect on the results. The influencing factors discovered during the test urge the continuous improvement of the standard.

In addition, China issued GB/T30865.1-2014 “Gloves for hand-held knife cuts and stab wounds” in 2014, which adopted the standards of ISO, IEC and other international organizations, but since March 23, 2017, this standard has been transformed into a recommended standard and is no longer mandatory.

## Summary and Outlook

In order to meet the growing needs of society, the field of security protection aims to obtain more flexible, functional and intelligent protective materials through the synthesis of high-performance fibers and the development of new modification technologies. Although the performance of raw fiber materials is the key to determining the cut-resistant performance of protective equipment, factors such as yarn structure, yarn linear density, fabric structure, and coating structure and performance are also closely related. Due to technical limitations such as raw material selection and processing methods, traditional cut-resistant textile materials still have certain limitations in terms of comfort and functionality. The continuous development of the synthesis and modification technology of various high-performance fibers has further overcome the shortcomings of traditional cut-resistant textile materials and expanded its application range.

Current cut-resistant equipment still has many shortcomings, such as heavy fabric, complex wearing steps, poor comfort, inconvenient movement. On the basis of meeting the requirements of safety protection, lightness, softness and comfort have become a new trend in the development of cut-resistant textiles. From the raw materials, preparation methods, modification processing, post-processing and application evaluation, etc., the cut-resistant textiles with better performance, lower cost and more complete categories should be developed. Chinese future cut-resistant textile industry needs to increase talent training, strengthen basic research and development, support key industries and key enterprises, formulate incentive policies and guarantee mechanisms that are conducive to the development of the industry, and promote the rapid development of the national cut-resistant textile industry (Jia and Li, 2020).
